# Radiological features of sinonasal NUT (nuclear protein in testis) carcinoma: case series and systematic review

**DOI:** 10.1007/s11604-025-01893-4

**Published:** 2025-10-25

**Authors:** Akira Baba, Shohei Kiso, Shotaro Naganawa, Satoshi Matsushima, Hideomi Yamauchi, Shu Okubo, Makoto Hinotsume, Yota Tabuchi, Kensaku Mori, Ashok Srinivasan, Hiroya Ojiri

**Affiliations:** 1 Department of Radiology, The Jikei University School of Medicine, 3-25-8 Nishi-Shimbashi, Minato-ku, Tokyo 105-8461 Japan; 2 Department of Radiology, The Cancer Institute Hospital of Japanese Foundation for Cancer Research, 3-8-31 Ariake, Koto-ku, Tokyo 135-8550 Japan; 3 Department of Radiology, University of California Davis Medical Center, 4860 Y Street, Suite 3100, Sacramento, CA 95817 USA; 4 Division of Neuroradiology, Department of Radiology, University of Michigan, 1500 E. Medical Center Dr., Ann Arbor, MI 48109 USA

**Keywords:** NUT carcinoma, Sinonasal, Computed tomography, Magnetic resonance imaging, Systematic review

## Abstract

**Purpose:**

NUT (nuclear protein in testis) carcinoma is a highly aggressive malignancy with poor prognosis, often leading to under-diagnosis due to limited recognition of its radiological features. This study aims to comprehensively analyze the radiological characteristics of sinonasal NUT carcinoma through a systematic review supplemented by institutional cases to facilitate early diagnosis and appropriate treatment planning.

**Materials and methods:**

A systematic review of MEDLINE, Scopus, and Embase databases was conducted following PRISMA 2020 guidelines to identify studies reporting CT and/or MRI features of sinonasal NUT carcinoma published up to May 29, 2024. Additional cases from our institution were included. Two board-certified radiologists with 15 and 18 years of experience jointly evaluated all images by consensus.

**Results:**

The study included 35 lesions from 35 patients (5 institutional, 30 from literature). Mean age was 37.3 years with equal sex distribution. The most commonly involved locations were ethmoid sinus (57.1%), nasal cavity (51.4%), and maxillary sinus (31.4%), with mean lesion diameter of 4.5 cm. On CT, all lesions demonstrated heterogeneous moderate enhancement, with calcification in 25.0% of cases. MRI revealed variable T2 signal intensities: mixed iso- and high signal (41.7%), high signal (33.3%), and mixed iso- and low signal (8.3%). Contrast-enhanced MRI showed heterogeneous moderate enhancement in 93.3% of cases. Necrotic areas were present in 48.1% of cases. Mean ADC value was 0.84 × 10⁻^3^ mm^2^/s. Invasive/destructive changes occurred in 91.4% of cases, with intraorbital extension in 52.9% and intracranial extension in 29.4%.

**Conclusion:**

Sinonasal NUT carcinoma demonstrates characteristic imaging features including predilection for ethmoid sinus and nasal cavity, heterogeneous enhancement, and extensive invasive/destructive changes with frequent orbital and intracranial extension. While these features overlap with other sinonasal malignancies, recognition of these patterns may facilitate earlier diagnosis of this rare but highly aggressive tumor.

## Introduction

NUT (nuclear protein in testis) carcinoma is a highly aggressive malignancy defined by a chromosomal rearrangement involving the NUT carcinoma family member 1 (NUTM1) gene on chromosome 15q14 [1]. Sinonasal NUT carcinoma is an exceptionally rare and aggressive tumor which arises from the epithelium of the sinonasal cavities, characterized by a poor prognosis, with most patients succumbing to the disease within 1 year of diagnosis [2]. Due to its rarity, this tumor has been under-reported in the literature and under-recognized in radiology, leading to frequent under-diagnosis and misdiagnosis [1]. Given its aggressive and rapidly progressive nature, a thorough understanding of its radiologic characteristics is crucial for facilitating early diagnosis and appropriate treatment planning. To date, most reports on sinonasal NUT carcinoma have been limited to isolated case reports and small case series, with no comprehensive analysis of its imaging features. Radiologic imaging plays a vital role in evaluating sinonasal tumors, especially in assessing their spatial extent, including intraorbital and intracranial invasion. It also provides supplementary information for tumor characterization. This study aims to provide a comprehensive summary of the existing literature through a systematic review, supplemented by a case series from our institution, with a focus on the CT and MRI characteristics of this rare entity.

## Methods

### Search strategy and study selection

**Fig. 1:**
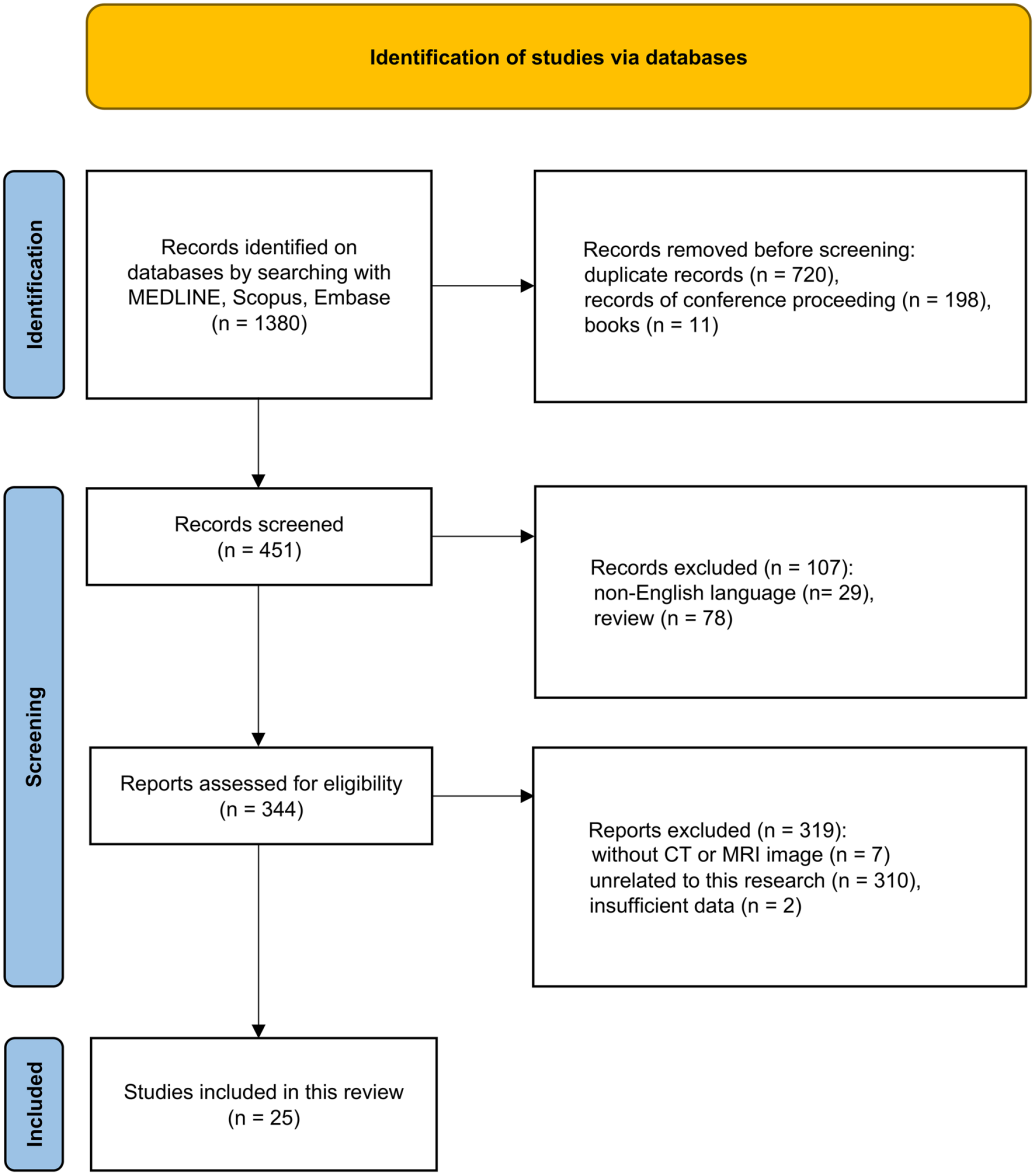
Flow diagram of the article selection process based on the Preferred Reporting Items for Systematic Reviews and Meta-analyses (PRISMA) 2020 guidelines. After applying the selection criteria, 25 eligible articles were identified. *n* number

MEDLINE (via PubMed), Scopus, and Embase databases were screened for a literature search of studies including CT and/or MR images of sinonasal NUT carcinoma published up to May 29, 2024 (Fig. 1). The search terms combinations were as follows:

(NUT carcinoma) OR (NUT midline carcinoma OR (nuclear protein in testis carcinoma) OR (nuclear protein in testis midline carcinoma) AND (head and neck OR sinonasal OR paranasal).

The eligibility criteria for the identified articles were as follows: 1) cases with pathologically confirmed diagnosis of primary sinonasal NUT carcinoma and 2) cases with CT and/or MR images. The exclusion criteria were as follows: 1) articles that were unrelated to sinonasal NUT carcinoma, 2) articles written in languages other than English, 3) lack of full-text availability, and 4) other types of records, including books and conference proceedings without a complete, peer-reviewed publication.

For the eligibility assessment, the exclusion of articles unrelated to this research and of case reports/series was performed by a board-certified radiologist with 15 years of experience in head and neck radiology, and the other eligibility assessments were jointly evaluated by consensus by two board-certified radiologists with 15 and 18 years of experience, respectively, in head and neck radiology.

In addition, we included five cases of sinonasal NUT carcinoma diagnosed at our institutions between 2014 and 2023. The Institutional Review Board waived the requirement for approval to include the findings from these five patients. These cases were identified using the electronic medical record search system. The search was conducted using the keywords ‘NUT’ and ‘Nuclear Protein in Testis.’ All identified cases were manually reviewed to confirm the diagnosis, and only those with histopathologically confirmed sinonasal NUT carcinoma and available pretreatment imaging were included. Cases with insufficient imaging data or ambiguous diagnoses were excluded.

This study was conducted in accordance with the Preferred Reporting Items for Systematic Reviews and Meta-Analyses (PRISMA) 2020 statement [3].

### Data analyses

Two board-certified radiologists with 15 and 18 years of experience, respectively, in head and neck radiology jointly evaluated all images by consensus. Demographic and clinical data were collected, including patient sex, age, presenting symptoms, treatment methods, presence of neck node involvement, distant metastasis, evidence of disease progression, progression-free survival duration, survival status, and follow-up duration.

The following radiological parameters were evaluated: location, laterality, size (longest diameter on any plane), predominant lesion density on non-contrast CT (NCCT) (in comparison with cerebral white matter on the same image plane), intralesional calcification on NCCT, enhancement pattern (homogeneous or heterogeneous) and degree on contrast-enhanced CT (CECT), signal intensities on T2-weighted imaging (T2WI) and T1-weighted imaging (T1WI) (in comparison with the cerebral white matter on the same image plane), enhancement pattern (homogeneous or heterogeneous) and degree on contrast-enhanced MRI (CEMRI)/post-gadolinium T1WI, apparent diffusion coefficient (ADC) values, necrotic non-enhancing change on CECT or CEMRI, invasive/destructive change in surrounding structures, intraorbital extension, and intracranial extension. The radiological findings were comprehensively analyzed based on both imaging and descriptive data.

For our institutional cohort of five patients, complete demographic and clinical data, along with comprehensive CT/MRI imaging, were available.

In contrast, cases reported in the literature were limited, with only partial demographic and clinical information, and incomplete imaging figures and corresponding descriptions with varying levels of detail. For cases retrieved from the literature, only studies that included published imaging figures were incorporated in our analysis. Imaging variables were basically extracted from these figures, and supplemented with textual descriptions when available.

### Quality assessment

Case-based studies were reviewed using a widely accepted tool proposed by Murad et al. [4], to evaluate the methodological quality of case reports and case series based on four domains (selection, ascertainment, causality, and reporting) and eight signaling questions. This tool has been widely applied in prior systematic reviews of case-based studies in head and neck radiology [5–9].

## Results

### Study selection

A total of 1380 articles were identified through the initial database search, of which 929 were excluded prior to screening. An additional 107 articles were excluded after the screening. Based on the eligibility assessment, a further 319 articles were excluded, resulting in 25 articles with 30 cases describing 30 lesions [10–34] **(**Fig. 1**)**. With the addition of five institutional cases (Table 1), the final study cohort comprised 35 patients with pathologically confirmed sinonasal NUT carcinoma involving 35 lesions.

**Table 1:** Demographic, clinical, and radiological data of the five patients in our institutions

Patients	1	2	3	4	5
Demographic and clinical data					
Age	41	42	45	51	46
Sex	Female	Male	Male	Male	Male
Presenting symptom	Eye pain	Headache	Periorbital swelling	Facial pain	Facial numbness
Treatment	CRT	Surgery + CRT	Surgery + CRT	Surgery + CRT	CRT
Neck node involvement	Yes	No	No	No	No
Distant metastasis	No	No	No	No	No
Progression disease	No	Yes	Yes	Yes	Yes
Progression-free survival duration (month)	3	6	24	1.5	3
Survival status	Death	Survive	Survive	Survive	Death
Follow-up duration (month)	3	8	120	8	6
Radiological data					
CT	Yes	Yes	Yes	Yes	Yes
MRI	Yes	Yes	Yes	Yes	Yes
Location	Ethmoid sinus	Ethmoid sinus + sphenoid sinus	Ethmoid sinus	Maxillary sinus	Maxillary sinus
Laterality	Right	Midline	Left	Right	Left
Size (cm)	5.2	2.9	5.9	4.7	6.2
Density on NCCT	NA	Intermediate and low	Intermediate	Intermediate	Intermediate
Calcification on NCCT	NA	No	Yes	No	No
Enhancement pattern on CECT	Heterogeneous	Heterogeneous	Heterogeneous	Heterogeneous	Heterogeneous
Enhancement degree on CECT	Moderate	Moderate	Moderate	Moderate	Moderate
Signal intensity on T2WI	High	High	High	Iso and high	High
Signal intensity on T1WI	Iso	Iso	Iso	Iso	Iso
Enhancement pattern on CEMRI	Heterogeneous	Heterogeneous	Heterogeneous	Heterogeneous	Heterogeneous
Enhancement degree on CEMRI	Intermediate	Slight	Intermediate	Intermediate	Intermediate
Mean ADC value (10^–3^ mm^2^/s)	0.64	1.08	NA	0.80	0.84
Invasive/destructive change in surrounding structures	Yes	Yes	Yes	Yes	Yes
Necrotic non-enhancing changes on CECT or CEMRI	Yes	No	Yes	Yes	Yes
Perineural spread	No	No	No	No	No
Intraorbital extension	Yes	No	Yes	No	Yes
Intracranial extension	No	No	Yes	No	No

*CRT* Chemoradiotherapy, *NA* Not available, *NCCT* Non-contrast CT, *CECT* Contrast-enhanced CT, *T2WI* T2-weighted image, *T1WI* T1-weighted image, *CEMRI* Contrast-enhanced MRI, *ADC* Apparent diffusion coefficient

### Risk of bias

*Selection*: Since we extracted data from case-based studies, the selection methods were rarely reported, which might have introduced selection bias.

*Ascertainment*: The number of analyzable cases varied in relation to the following parameters: sex (29/30, 96.7%), presenting symptoms (27/30, 90%), treatment method (25/30, 83.3%), neck node involvement (28/30, 93.3%), distant metastasis (28/30, 93.3%), evidence of disease progression (29/30, 96.7%), progression-free survival duration (22/30, 73.3%), survival status (29/30, 96.7%), follow-up duration (28/30, 93.3%), size (7/30, 23.3%), density on NCCT (5/30, 16.7%), calcification on NCCT (4/30, 13.3%), enhancement pattern on CECT (15/30, 50.0%), enhancement degree on CECT (15/30, 50.0%), signal intensities on T2WI (7/30, 23.3%), signal intensities on T1WI(4/30, 13.3%), enhancement pattern on CEMRI (9/30, 30.0%), enhancement degree on CEMRI (10/30, 33.3%), ADC value (0/30, 0%), necrotic non-enhancing change (22/30, 73.3%), intraorbital extension (29/30, 96.7%), and intracranial extension (29/30, 96.7%).

*Causality*: The progression-free survival duration of the patients ranged from 1 to 34 months and follow-up duration of the patients ranged from 3 to 34 months.

*Reporting*: In most cases, not all relevant CT and MRI sequences were performed or reported.

### Demographic data

Demographic and clinical data are summarized in Table 2. The mean age at diagnosis was 37.3 years (range, 0.75–84 years), with an equal sex distribution (17:17). The most common presenting symptom was nasal obstruction (13/32, 40.6%) followed by epistaxis (8/32, 25.0%). Most patients underwent surgery combined with chemoradiotherapy (CRT) (16/35, 45.7%) or CRT alone (12/35, 34.3%). Neck node involvement was present in 24.2% (8/33) of patients, and distant metastases were observed in 21.2% (7/33). Disease progression was observed in 64.7% (22/34) of patients. The mean progression-free survival duration was 8.6 months (range: 1–34 months). At the time of analysis, 55.9% (19/34) of patients were deceased. The mean follow-up duration was 13.9 months (range, 3–120 months).

**Table 2:** Demographic and clinical information of the 35 patients

Demographic	
Mean age at diagnosis (years [range]) (*N* = 35)	37.3 [0.75–84]
Sex (*N* = 34)	Male = 17, Female = 17
Clinical	
Presenting symptoms (*N* = 32)	
Nasal obstruction	13 (13/32, 40.6%)
Epistaxis	8 (8/32, 25.0%)
Facial pain	6 (6/32, 18.8%)
Eye pain	5 (5/32, 15.6%)
Lacrimation	5 (5/32, 15.6%)
Headache	5 (5/32, 15.6%)
Diplopia	5 (5/32, 15.6%)
Nasal discharge	5 (5/32, 15.6%)
Visual disturbance	4 (4/32, 12.5%)
Periorbital swelling	3 (3/32, 9.4%)
Nasal pain	3 (3/32, 9.4%)
Facial numbness	2 (2/32, 6.3%)
Bloody nasal discharge	2 (2/32, 6.3%)
Frontal swelling	2 (2/32, 6.3%)
Cheek swelling	2 (2/32, 6.3%)
Tinnitus	1 (1/32, 3.1%)
Hearing loss	1 (1/32, 3.1%)
Nasal swelling	1 (1/32, 3.1%)
Olfactory disorder	1 (1/32, 3.1%)
Patients with multiple symptoms (*N* = 32)	19 (19/32, 59.4%)
Treatment (*N* = 35)	
Surgery combined with CRT	16 (16/35, 45.7%)
CRT	12 (12/35, 34.3%)
RT	2 (2/35, 5.7%)
Surgery combined with chemotherapy	1 (1/35, 2.9%)
Neck node involvement (*N* = 33)	8 (8/33, 24.2%)
Distant metastasis (*N* = 33)	7 (7/33, 21.2%)
Progression disease (*N* = 34)	22 (22/34, 64.7%)
Mean progression-free survival duration (months [range]) (*N* = 34)	8.6 (1–34)
Survival status (*N* = 34)	
Death	19 (19/34, 55.9%)
Mean follow-up duration (months [range]) (*N* = 34)	13.9 (3–120)

*N* Number of patients, *CRT* Chemoradiotherapy, *RT* Radiotherapy

### Radiological findings

Radiological findings are summarized in Table 3. Involvement of multiple sinonasal sites was observed in over half of the cases (19/35, 54.3%). The most common location of involvement was the ethmoid sinus (57.1%, 20/35), followed by the nasal cavity (51.4%, 18/35), and maxillary sinus (31.4%, 11/35). The lesions were right-sided in 44.1% (15/34), left-sided in 29.4% (10/34), and midline in 26.5% (9/34). The mean lesion diameter was 4.5 cm (range, 2.5−6.2 cm).

**Table 3:** Radiological characteristics of the 35 lesions

Imaging modality	
CT	26 (22/35, 62.9%)
MRI	19 (19/35, 54.3%)
Parameters	
Multiple location involvement (*N* = 35)	19 (19/35, 54.3%)
Location involvement (*N* = 35)	
Ethmoid sinus	20 (20/35, 57.1%)
Nasal cavity	18 (18/35, 51.4%)
Maxillary sinus	11 (11/35, 31.4%)
Sphenoid sinus	8 (8/35, 22.9%)
Frontal sinus	4 (4/35, 11.4%)
Laterality (*N* = 34)	
Right	15 (15/34, 44.1%)
Left	10 (10/34, 29.4%)
Midline	9 (9/34, 26.5%)
Mean size (cm [range]) (*N* = 12)	4.5 (2.5–6.2)
Density on NCCT (*N* = 9)	
Iso	8 (8/9, 88.9%)
Iso and low	1 (1/9, 11.1%)
Calcification on NCCT (*N* = 8)	
Yes	2 (2/8, 25.0%)
No	5 (5/8, 75.0%)
Enhancement pattern on CECT (*N* = 21)	
Heterogeneous	21 (21/21, 100%)
Enhancement degree on CECT (*N* = 21)	
Moderate	21 (21/21, 100%)
Signal intensities on T2WI (*N* = 12)	
Iso and high	5 (5/12, 41.7%)
High	4 (4/12, 33.3%)
Iso and low	1 (1/12, 8.3%)
Signal intensities on T1WI (*N* = 9)	
Iso	6 (6/9, 66.7%)
Iso and low	3 (3/9, 33.3%)
Enhancement pattern on CEMRI (*N* = 15)	
Heterogeneous	14 (14/15, 93.3%)
Homogeneous	1 (1/15, 6.7%)
Enhancement degree on CEMRI (*N* = 15)	
Moderate	14 (14/15, 93.3%)
Slight	1 (1/15, 6.7%)
Mean ADC value (range) (10^−3^ mm^2^/s) (*n* = 4)	0.84 (0.64–1.08)
Invasive/destructive change in surrounding structures	
Yes	32 (32/35, 91.4%)
No	3 (3/35, 8.6%)
Necrotic non-enhancing changes on CECT or CEMRI (*N* = 27)	
Yes	13 (13/27, 48.1%)
No	14 (14/27, 51.9%)
Perineural spread (*N* = 34)	
No	34 (34/34, 100%)
Intraorbital extension (*N* = 34)	
Yes	18 (18/34, 52.9%)
No	16 (16/34, 47.1%)
Intracranial extension (*N* = 34)	
Yes	10 (10/34, 29.4%)
No	24 (24/34, 70.6%)

*N* Number of patients, *NCCT* Non-contrast computed tomography, *CECT* Contrast-enhanced computed tomography, *T2WI* T2-weighted imaging, *T1WI* T1-weighted imaging, *CEMRI* Contrast-enhanced magnetic resonance imaging, *ADC* Apparent diffusion coefficient

On NCCT, most lesions exhibited isodense (88.9%, 8/9), with one case showing a combination of iso- and low density (11.1%, 1/9). Calcification was observed in 25.0% (2/8) of cases. All lesions demonstrated heterogeneous moderate enhancement on CECT (100%, 21/21).

On MRI, signal intensities on T2WI were variable, with mixed iso- and high signal in 41.7% (5/12), high signal in 33.3% (4/12), and iso- and low signal in 8.3% (1/12) of cases. On T1WI, most lesions exhibited isointensity (66.7%, 6/9), while 33.3% (3/9) showed mixed iso- and low signal. The enhancement on CEMRI was predominantly heterogeneous (93.3%, 14/15), with a moderate degree of enhancement in most cases (93.3%, 14/15). The mean ADC value was 0.84 × 10^−3^ mm^2^/s (range: 0.64–1.08 × 10^−3^ mm^2^/s) in the four measured cases.

**Fig. 2:**
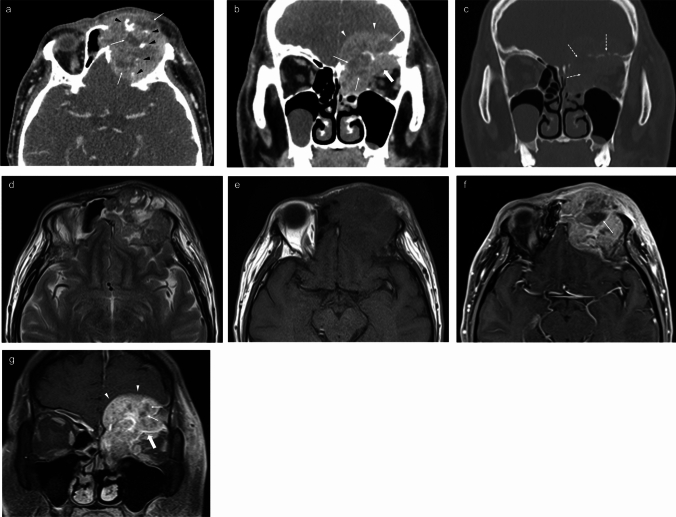
Sinonasal nuclear protein in testis carcinoma (case 3). Contrast-enhanced CT axial image (**a**) and coronal image (**b**) show a tumor in the left ethmoid sinus with heterogeneous moderate enhancement, with a necrotic area inside (white arrow) and irregular calcification (black arrowheads). The lesion demonstrates intracranial extension via destruction of the skull base and left supraorbital wall (arrowheads) and intraorbital extension via destruction of the medial lateral orbital wall (bold arrows). Bone algorithm CT coronal image (**c**) shows absence of the skull base and medial orbital wall, indicating bone destruction (dotted arrows). The solid component of the lesion shows high signal intensity in the T2-weighted image (**d**) and isointense signal on T1-weighted image (**e**). Post-contrast T1 axial (**f**) and coronal (**g**) images show heterogeneous moderate enhancement of the tumor, with internal necrotic areas (arrows), intracranial extension (arrowheads) and intraorbital extension (bold arrows)

**Fig. 3:**
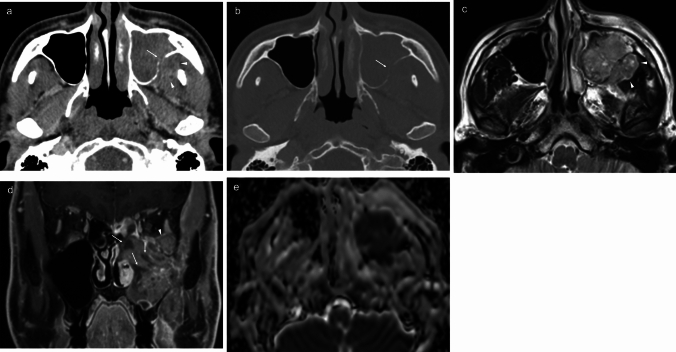
Sinonasal nuclear protein in testis carcinoma (case 5). Contrast-enhanced CT axial image (**a**) and bone algorithm CT axial image (**b**) shows a tumor with heterogeneous moderate enhancement in the left maxillary sinus, with destructive changes in the posterior wall of the left maxillary sinus (arrows) and tumor extension into the left retroantral fat pad (arrowhead). T2-weighted axial image (**c**) shows high signal intensity lesion with tumor extension to the left retroantral fat pad (arrowhead). Post-contrast T1 coronal image (**d**) shows heterogeneous moderate enhancement of the lesion, with an internal necrotic area (arrows) and intraorbital extension (arrowhead). ADC map (**e**) demonstrates a value of 0.84 × 10⁻^3^ mm^2^/s in the solid portion of the tumor

**Fig. 4:**
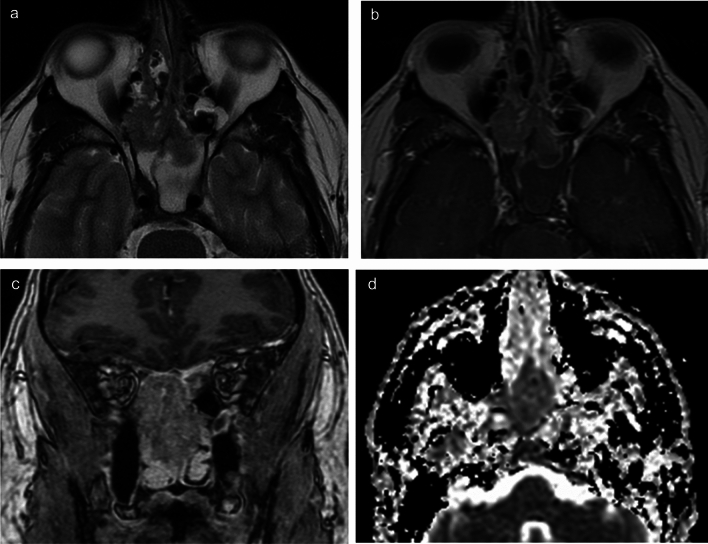
Sinonasal nuclear protein in testis carcinoma (case 2). T2-weighted axial image (**a**) shows a tumor with slightly high signal intensity extending from the right ethmoid sinus to the bilateral sphenoid sinuses. Post-contrast T1 axial image (**b**) and coronal images (**c**) show heterogeneous slight enhancement. The lesion is confined to the paranasal sinuses with no surrounding invasive or destructive changes, no intracranial extension, and no intraorbital extension. ADC map (**d**) demonstrates a value of 1.08 × 10⁻^3^ mm^2^/s within the lesion

Destructive changes to adjacent structures were observed in 91.4% (32/35) of cases. Necrotic, non-enhancing changes on CT or MRI were identified in 48.1% (13/27) of cases. Perineural spread was absent in all evaluated cases on CT or MRI (0%, 0/34). Intraorbital extension was observed in 52.9% (18/34) and intracranial extension in 29.4% (10/34). Three representative patients are shown in Figs. 2, 3, and 4.

## Discussion

This study presents a comprehensive analysis of the radiological characteristics of sinonasal NUT carcinoma, combining data from a systematic review of the existing literature with five additional cases from our institution. To our knowledge, this is the first and largest analytical study focusing on the radiological features of sinonasal NUT carcinoma.

The clinical features of sinonasal NUT carcinoma observed in our study included a broad age range, consistent with previous reports [35–37]. This finding underscores that the tumor can affect patients across a wide age spectrum, including both young and elderly individuals. In our study, no sex difference was observed, which is consistent with previous reports [36]. These results suggest that sinonasal NUT carcinoma should be considered in patients across a broad demographic spectrum. The predominant symptoms were nasal obstruction and epistaxis, both of which are nonspecific, highlighting the challenge of early clinical diagnosis and the importance of diagnostic imaging.

The most common treatment approach identified in the study results was a combination of surgery and CRT, followed by CRT alone. The prognosis was poor, with a mean progression-free survival of 8.6 months, disease progression observed in over half of the patients, a mortality rate exceeding 50%, and a mean follow-up duration of approximately 1 year. This finding is consistent with previous reports that have described the poor prognosis of this disease [35, 36]. These findings demonstrate the aggressive clinical behavior and high malignant potential of NUT carcinoma, underscoring the importance of early diagnosis and a multidisciplinary treatment approach. Disease management is further complicated by the presence of neck lymph node metastases and distant metastases in a significant proportion of cases.

Radiological findings in this study demonstrated that the lesions were predominantly located in the ethmoid sinus, nasal cavity, and maxillary sinus, in decreasing order of frequency. NUT carcinoma was formerly referred to as “NUT midline carcinoma,” a term that reflects its typical midline localization [26, 30, 37]. The predilection for the ethmoid sinus and nasal cavity, which are located relatively midline in the sinonasal sinus region, may be indicative of the disease’s characteristic features. Regarding lesion laterality, the right side was most common, followed by the left side and the midline locations, though lesions were not always strictly confined to the absolute midline. The tumor diameter was substantial, indicating that these lesions had already reached a considerable tumor size by the time of diagnosis.

CT findings in this study revealed that all lesions demonstrated heterogeneous moderate enhancement with contrast, likely reflecting the histological heterogeneity typical of sinonasal NUT carcinoma.

MRI findings in this study demonstrated greater variability. On T2WI, several patterns were observed, including mixed iso- and high signal intensities, high signal intensity alone, and mixed iso- and low signal intensities. CEMRI showed heterogeneous moderate enhancement in most cases. These findings likely reflect the underlying histological heterogeneity of the tumor. Necrotic non-enhancing areas were present in many cases, likely indicating inadequate blood supply resulting from rapid tumor growth. ADC values derived from DWI have been reported to be significantly lower for malignant head and neck lesions compared to benign or inflammatory lesions [38–40], making DWI a useful MR sequence that reflects the pathological characteristics of head and neck tumors. In this study, the mean ADC value across four cases was 0.84 × 10^–3^ mm^2^/s, which is similar to or slightly lower than previously reported values for sinonasal squamous cell carcinoma (mean ADC value of 0.90 × 10–3 mm2/s in 59 patients) [41–43] and may reflect the hypercellularity characteristic of sinonasal NUT carcinoma.

Invasive and destructive changes to surrounding tissues were observed in most of cases, with intraorbital extension in approximately half and intracranial extension in nearly a third of cases. In addition, involvement of multiple sinonasal sites was observed in over half of the cases. These findings suggest that certain imaging features may aid in distinguishing sinonasal NUT carcinoma from other malignancies, though further comparative studies are needed to validate these distinctions.

While sinonasal NUT carcinoma exhibits certain characteristic radiologic features, it also shares several imaging similarities with other malignant sinonasal tumors, including sinonasal squamous cell carcinoma (SCC), sinonasal undifferentiated carcinoma (SNUC), and SMARCB1-deficient sinonasal carcinoma, making radiological differentiation challenging [44, 45]. Common imaging characteristics, including heterogeneous contrast enhancement, infiltrative growth patterns, and necrotic areas, are observed across these entities, hampering definitive differentiation based solely on imaging features. While integrating clinical, radiologic, and pathologic data are crucial for raising suspicion of sinonasal NUT carcinoma, definitive diagnosis ultimately relies on histopathological confirmation with specific immunohistochemical testing. Given this tumor’s rarity, raising awareness and improving recognition of its imaging features are critical steps toward reducing diagnostic delays and potentially improving patient outcomes.

As highlighted in this study, MRI plays a pivotal role in evaluating sinonasal tumors by assessing their spatial extent, particularly invasive features such as orbital and intracranial extension. This aligns with the consensus that MRI’s primary function is to delineate the spatial infiltration of head and neck tumors and clarify their relationships with adjacent critical structures [46–48]. While our study also analyzed imaging features such as T2 signal intensity and ADC values, these imaging findings should be considered complementary to histopathological diagnosis rather than definitive diagnostic markers on their own.

Several limitations of this study should be acknowledged. First, the exceedingly limited sample size constrains the generalizability of our results. Second, MRI was available in only approximately half of cases, potentially impacting our comprehensive assessment of imaging characteristics. This limitation further restricts the generalizability of our findings, particularly regarding the complete radiologic spectrum of sinonasal NUT carcinoma. While the sample size is small, this reflects the extreme rarity of sinonasal NUT carcinoma in clinical practice. Despite this limitation, our study provides a critical foundation for understanding the radiologic characteristics of this malignancy, which remains under-reported and under-recognized in the literature. The retrospective nature of case series and systematic reviews introduces potential biases, including selection bias and reporting bias, particularly since not all studies provided comprehensive radiological or clinical data. Some imaging features analyzed (size, density on NCCT, calcification on NCCT, signal intensity on T1WI) have notably limited sample sizes due to incomplete descriptions in the literature cases. Variations in imaging techniques and a lack of standardized reporting further contribute to potential discrepancies in the interpretation of radiological findings. Furthermore, the absence of a comparative analysis with sinonasal SCC represents an additional limitation of this study. While such a comparison would provide valuable insights into the imaging characteristics distinguishing sinonasal NUT carcinoma from the more common SCC, it was not feasible due to incomplete clinical and radiological data available for many included cases. Future multicenter studies incorporating consecutive cases with comprehensive datasets are needed to explore these differences more thoroughly. Given these limitations, the results of this study should be interpreted with appropriate caution.

## Conclusion

This systematic review combined with institutional cases represents the largest analysis of sinonasal NUT carcinoma imaging characteristics to date. Our findings demonstrate that sinonasal NUT carcinoma exhibits characteristic imaging features, including predilection for ethmoid sinus and nasal cavity, heterogeneous enhancement, and extensive invasive/destructive changes with frequent orbital and intracranial extension. While these features overlap with other sinonasal malignancies, they provide important baseline data for radiological recognition. Although the rarity of this tumor limits comprehensive analysis, these findings provide crucial insights that could facilitate earlier recognition and underscore the importance of maintaining high clinical suspicion for NUT carcinoma in the differential diagnosis of aggressive sinonasal masses.
